# Supplementary material for: The denitrosylase SCoR2 controls cardioprotective metabolic reprogramming
Source: bioRxiv. 2025 Jul 18:2025.03.12.642752. Originally published 2025 Mar 14. Preprint. [Version 2] doi: 10.1101/2025.03.12.642752 (PMC11952481; doi:10.1101/2025.03.12.642752)
Supplement: 4 [file NIHPP2025.03.12.642752v2-supplement-4.pdf]

bioRxiv preprint doi: https://doi.org/10.1101/2025.03.12.642752; this version posted July 18, 2025. The copyright holder for this preprint (which was not certified by peer review) is the author/funder, who has granted bioRxiv a license to display the preprint in perpetuity. It is made available under aCC-BY 4.0 International license.

Supplementary Materials

Figure S1: Characterization of SCoR2$^{-/-}$ mice at baseline and 24 hr post-MI.

Figure S2: Characterization of SCoR2 and protein S-nitrosylation in SCoR2$^{-/-}$ mice.

Figure S3: Characterization of BDH1 S-nitrosylation at the conserved SNO site Cys^{115}.

bioRxiv preprint doi: https://doi.org/10.1101/2025.03.12.642752; this version posted July 18, 2025. The copyright holder for this preprint (which was not certified by peer review) is the author/funder, who has granted bioRxiv a license to display the preprint in perpetuity. It is made available under aCC-BY 4.0 International license.

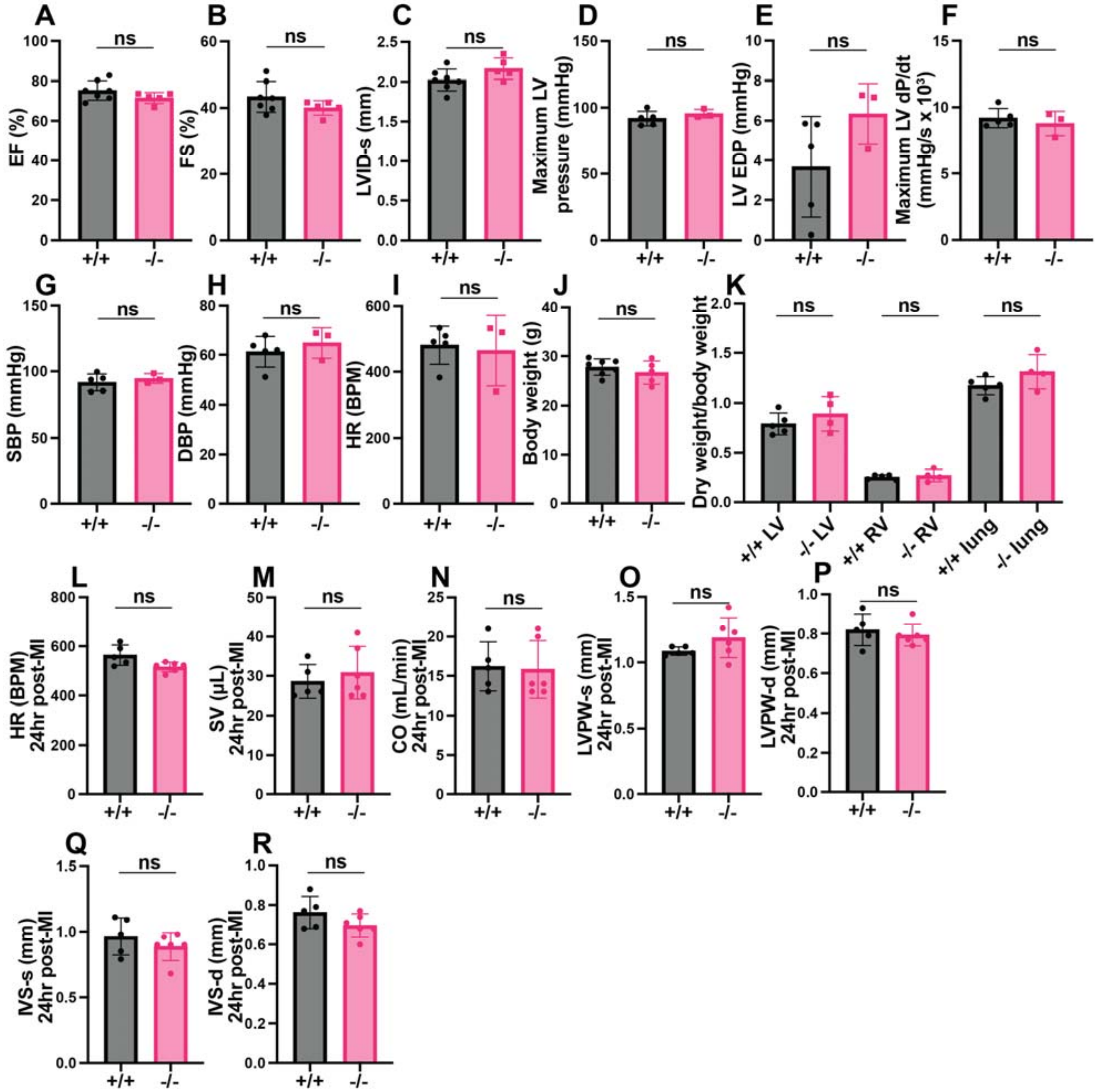

Figure S1. Characterization of SCoR2$^{-/-}$ mice at baseline and 24 hours post-MI. (**A-K**) Quantification of left ventricular contractile function (**A-F**), blood pressure (**G,H**), heart rate (**I**), and weight (**J,K**) in uninjured SCoR2$^{+/+}$ (+/+) and SCoR2$^{-/-}$ (–/–) mice at baseline. Ejection fraction (EF; (A)), fractional shortening (FS; (B)), left ventricular diameter at end systole (LVID-s; (C)), maximum LV pressure (D), left ventricular end diastolic pressure (LV EDP; (E)), maximum change in LV pressure over time (LV dP/dt; (F)), systolic blood pressure (SBP; (G)), diastolic blood pressure (DBP; (H)) and heart rate (HR; (I)) were determined by

bioRxiv preprint doi: https://doi.org/10.1101/2025.03.12.642752; this version posted July 18, 2025. The copyright holder for this preprint (which was not certified by peer review) is the author/funder, who has granted bioRxiv a license to display the preprint in perpetuity. It is made available under aCC-BY 4.0 International license.

echocardiography in N=3-7 uninjured +/+ and –/– mice. Body weight and ratio of left ventricle (LV), right ventricle (RV), and lung dry weight to body weight were quantified in N=4-5 +/+ and –/– mice in (J) and (K), respectively. At 24 hr post-MI, heart rate (HR; (**L**)) and left ventricular parameters (stroke volume (SV; (**M**)), cardiac output (CO; (**N**)), left ventricular posterior wall at end systole/diastole (LVPW-s (**O**), LVPW-d; (**P**)) and interventricular septal at end systole/diastole (IVS-s (**Q**), IVS-d (**R**))) were determined by echocardiography in N=5-6 +/+ and –/– mice. Statistical significance in (A-R) was determined by two-tailed Mann-Whitney test; ns = not significant.

bioRxiv preprint doi: https://doi.org/10.1101/2025.03.12.642752; this version posted July 18, 2025. The copyright holder for this preprint (which was not certified by peer review) is the author/funder, who has granted bioRxiv a license to display the preprint in perpetuity. It is made available under aCC-BY 4.0 International license.

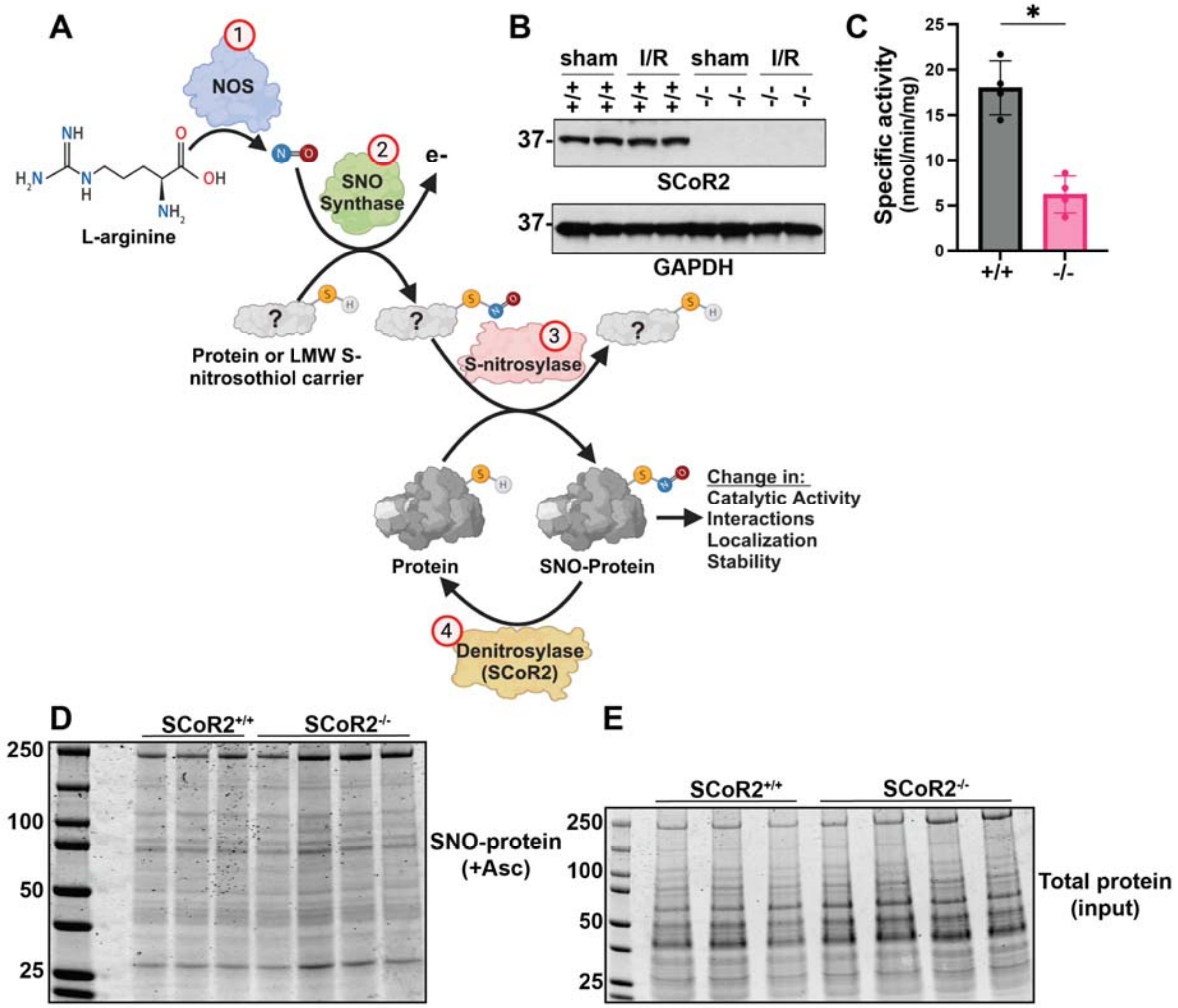

Figure S2. Characterization of SCoR2 and protein S-nitrosylation in SCoR2$^{-/-}$ mice. (**A**) Protein S-nitrosylation is regulated by four distinct classes of enzymes; NOS enzymes synthesize NO from L-arginine (1), SNO synthase enzymes produce S-nitrosothiols (SNOs) from NO (2), S-nitrosylase (aka transnitrosylase) enzymes transfer SNO to target proteins to regulate activity, interactions, localization or stability (3), denitrosylase enzymes (such as SCoR2) remove SNO from substrate proteins (4). Image created with Biorender.com. (**B**) Representative Western blot showing expression of SCoR2 in hearts of +/+ and –/– mice after sham or ischemia-reperfusion (I/R) surgery. N=4 mice per genotype. (**C**) NADPH-dependent SNO-CoA reductase activity measured in heart extracts from healthy untreated +/+ and –/– mice; N=4 mice per genotype. (**D,**

bioRxiv preprint doi: https://doi.org/10.1101/2025.03.12.642752; this version posted July 18, 2025. The copyright holder for this preprint (which was not certified by peer review) is the author/funder, who has granted bioRxiv a license to display the preprint in perpetuity. It is made available under aCC-BY 4.0 International license.

E) SNO-proteins identified via SNORAC from heart tissue isolated from healthy untreated +/+ (N=3) and –/– (N=4) mice. SNO-proteins shown in +Asc gel (D), total protein identified via Coomassie staining in input gel (E). Statistical significance in (C) determined by two-tailed Mann-Whitney test; * $p \leq 0.05$.

bioRxiv preprint doi: https://doi.org/10.1101/2025.03.12.642752; this version posted July 18, 2025. The copyright holder for this preprint (which was not certified by peer review) is the author/funder, who has granted bioRxiv a license to display the preprint in perpetuity. It is made available under aCC-BY 4.0 International license.

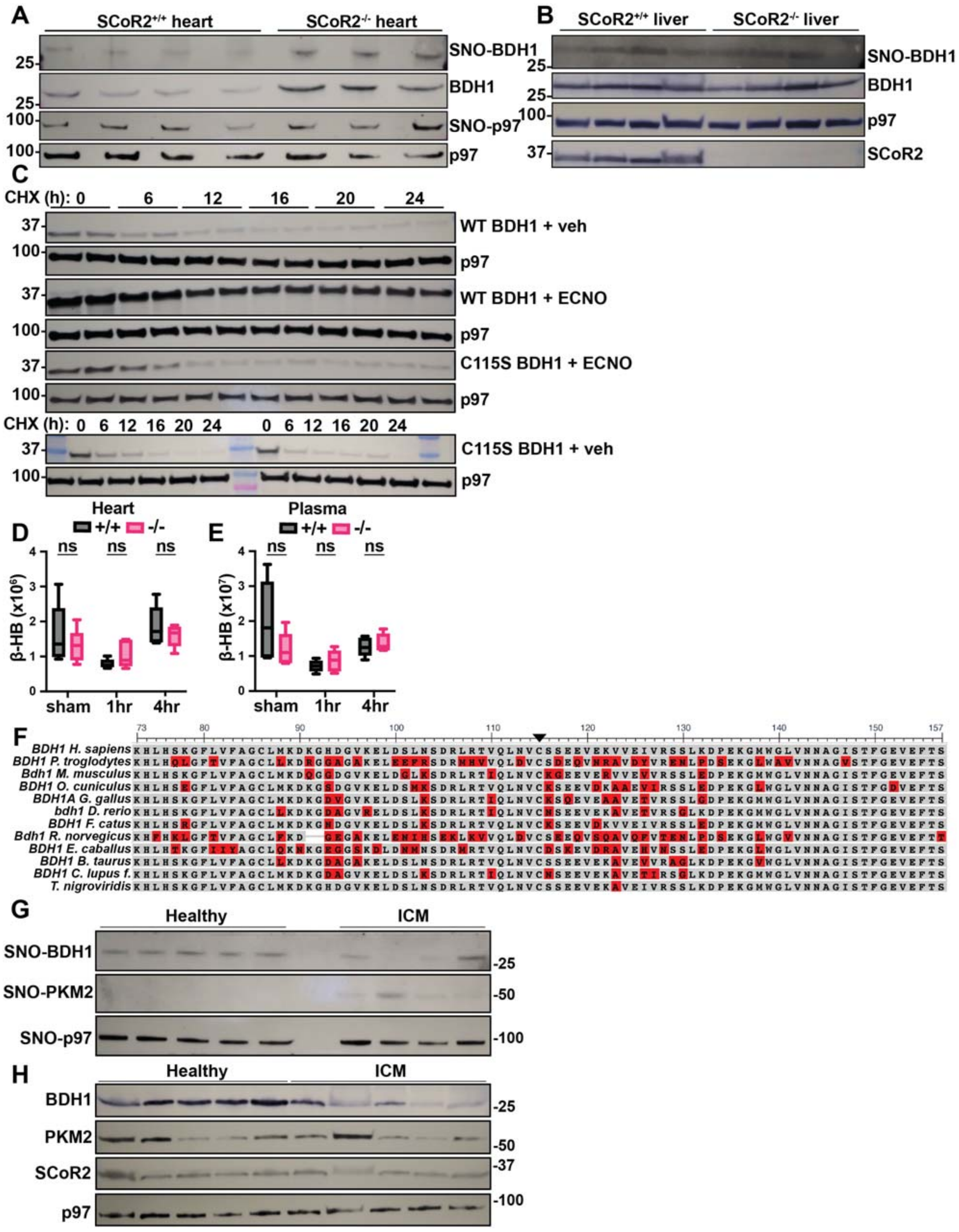

Figure S3. Characterization of BDH1 S-nitrosylation at conserved SNO site Cys^{115}. (A)

bioRxiv preprint doi: https://doi.org/10.1101/2025.03.12.642752; this version posted July 18, 2025. The copyright holder for this preprint (which was not certified by peer review) is the author/funder, who has granted bioRxiv a license to display the preprint in perpetuity. It is made available under aCC-BY 4.0 International license.

Representative gel from SNORAC measuring SNO-BDH1 and Western blot measuring total BDH1, relative to p97 ATPase loading control, in mouse heart (4 hr reperfusion), quantified in Fig. 4A-C. (**B**) SNO-BDH1 assessed by SNORAC in SCoR2$^{+/+}$ (+/+) and SCoR2$^{-/-}$ (–/–) liver tissue (N=4 each) from mice (1hr reperfusion), quantified in Fig. 4D-F. (**C**) Representative Western blot, quantified in Fig. 4H,I, denoting a CHX pulse-chase assay, in which HEK293 cells transfected with V5-tagged BDH1 were treated with 100µg/mL CHX to block new protein synthesis, together with 100µM ECNO or vehicle, with samples collected at 6-24hr. Duplicates are shown by treatment condition. (**D,E**) Beta-hydroxybutyrate (β-HB) quantified as ion abundance in +/+ and –/– mouse heart and plasma by LC/MS-based untargeted metabolite profiling, N=5 mice per condition per genotype. (**F**) Sequences of BDH1, or protein product of closest homology, from selected species aligned with constraint-based multiple alignment tool (COBALT); red color shows differences from the *H. sapiens* BDH1 sequence. Cys^{115} is indicated by black arrow. *T. nigroviridis* protein product ID is CAG04267. (**G,H**) Representative gel from SNORAC assessing SNO-BDH1 and SNO-PKM2 expression relative to SNO-p97 ATPase loading control (G) and from Western blot assessing BDH1, PKM2, and SCoR2 expression relative to p97 ATPase loading control (H) in human hearts without (N=10) and with (N=8-9) diagnosis of ischemic cardiomyopathy (ICM) (IRB # Pro00005621). Statistical significance in (D, E) determined by independent Student's t-tests performed between genotypes at each time-point.
